# The utilisation of regulated standardised care packages by Danish chiropractors: a mixed methods study

**DOI:** 10.1186/s12998-022-00423-7

**Published:** 2022-03-08

**Authors:** Rikke Krüger Jensen, Inge Ris, Elisabeth Linnebjerg, Henrik Wulff Christensen, Corrie Myburgh

**Affiliations:** 1 Department of Sport Science and Clinical Biomechanics, University of Southern Denmark, Campusvej 55, 5230 Odense M, Denmark; 2 Chiropractic Knowledge Hub, Campusvej 55, 5230 Odense M, Denmark; 3 Univerity College Lillebaelt, Niels Bohrs Allé 1, 5230 Odense M, Denmark

**Keywords:** Chiropractor, Standardised care package, Determinants of implementation behaviour questionnaire

## Abstract

**Background:**

In Denmark, chiropractors in primary care work as independent private contractors regulated by the Danish National Health Authorities. The regulation includes partial reimbursement intended for standardised care packages for lumbar and cervical radiculopathy and lumbar spinal stenosis. Random checks have shown lower use than expected. This study aimed to describe and explore the utilisation of standardised chiropractic care packages and identify barriers to uptake.

**Methods:**

A convergent mixed-method design was conceptualised. The use of standardised care packages was collected by register data. Potential determinants of difference in utilisation were assessed using a modified version of the Determinants of Implementation Behaviour Questionnaire (DIBQ) divided into 13 domains and sent to chiropractors in private clinics in Denmark in 2019. An open-ended question was added to the questionnaire, and thematic content analysis was applied. Qualitative findings were used to expand on the DIBQ data providing further insight into the clinicians’ perspective on standardised care packages.

**Results:**

Registry data of 244 included chiropractic clinics showed limited and inconsistent use of the standardised chiropractic care packages. A total of 269 chiropractors (44%) answered the DIBQ, and 45 provided data for the qualitative analyses. At least 60% of the clinicians answered ‘Strongly agree’ or ‘Agree’ in 10 out of 13 DIBQ domains suggesting a positive attitude towards using the standardised care packages. Three domains were identified as ‘problematic’ as more than 20% of clinicians disagreed or strongly disagreed: ‘Socio-political context’, ‘Goals’ and ‘Innovation’. Qualitative findings indicated that lack of usage of the standardised care packages was mainly related to the practical organization of standardised care, the chiropractor’s role when managing patients, and the patient population of interest to the clinic (e.g., children, athletes).

**Conclusion:**

In general, Danish chiropractors displayed positive attitudes towards standardised packages of care. However, considerable variation in the use of the standardised care programs was observed. Low utilisation seemed mainly related to logistics, the chiropractor’s role, collaboration with GPs, and the patient population of interest to the clinic. These findings should be further explored in more extensive qualitative studies to inform implementation initiatives to increase and rectify utility.

**Supplementary Information:**

The online version contains supplementary material available at 10.1186/s12998-022-00423-7.

## Background

Healthcare providers commonly work in healthcare systems regulated by professional bodies and government organisations. Regulations empower the government to shape the behaviour of service providers in primary health care practice settings by allowing it to maintain the quality and safety of care offered by health professionals and to control the market in healthcare services [1]. Regulation can include health care packages ensuring that clinicians deliver services in agreement with current national guidelines on clinical excellence. Health care packages are designed to direct resources to priority areas of health service delivery, and often describe specific services to ensure quality and consistency in patient management.

In Denmark, chiropractors in primary care work as independent private contractors regulated by the Danish National Health Authorities. The terms of regulation include partial reimbursement by the National Health Care intended for three standardised care packages for lumbar spinal stenosis, lumbar radiculopathy, and cervical radiculopathy, respectively [2]. They were developed to help and support professional standards without inhibiting daily clinical activities and describe a management structure and logistics of the patients’ care pathway that the chiropractors are obligated to comply with. Management includes time-fixed follow-up sessions to monitor the progression of symptoms. A time-fixed consultation includes case history and clinical examination related to the specific diagnosis, reassessment of the treatment plan based on the patient’s status, medical record-keeping, and standardised written communication with the patient’s general practitioner (GP). The content of the program is adherent to national clinical guidelines [3–5].

The patient reimbursement at an initial consultation is 16% for a common musculoskeletal problem such as non-specific low back pain. However, if the patient is diagnosed with cervical or lumbar radiculopathy or lumbar spinal stenosis and is admitted to a standardised care package, the reimbursement is 60%. Time-fixed follow-up sessions and additional treatment sessions reimburse 40% for patients with these specific conditions compared to 9–18% for patients in common care pathways. The fee for the patient is thereby reduced significantly. The benefit for the chiropractor is the increased recognition of the quality of services provided and the increased patient flow into chiropractic clinics. The incentive is, therefore, direct for the patient (less out-of-pocket expense) and indirect for the chiropractor (quality of services and more guaranteed business).

To increase knowledge of the content and administration of the standardised care packages, several implementation activities were initiated in 2017 and 2018, including information meetings, publications in a professional journal for chiropractors, newsletters per e-mails, booklets for clinicians and patients, podcasts, and personal contact to the clinics with very low or no use of standardised care packages [6].

However, although chiropractors who receive reimbursement from the national health care system are obliged to comply with the collective agreement on specific chiropractic services, the variation in usage between clinics indicated that not all chiropractors had embraced the new care packages. Statistics from 2017 and 2018 showed that the standardised care packages were used approximately 40% less than expected based on the estimation of approximately 15,000 patients yearly with relevant diagnosis and that there was a wide variety of usage between the individual chiropractic clinics [6, 7].

It stands to reason that hidden barriers could exist, resulting in lower-than-expected levels of use. Most patients with disc herniation or lumbar spinal stenosis can be managed in primary care, and only a few require referral for evaluation in secondary care with is both more expensive and less flexible than primary care [8, 9]. The application of standardised care packages among Danish chiropractors is a case describing some of the challenges with regulating private independent health care professionals.

Therefore, this study aimed to describe and explore the utilisation of standardised chiropractic care packages from 2017 to 2020. To operationalize the study aim, we posed three questions:

1. How did the use of standardised chiropractic care packages develop from 2017 to 2020?
2. What clinician-behavioural determinants influenced the utilisation of standardised care packages?
3. Can clinicians’ perspectives on utility processes help explain their behaviours?

## Method

### Design

**Fig. 1:**
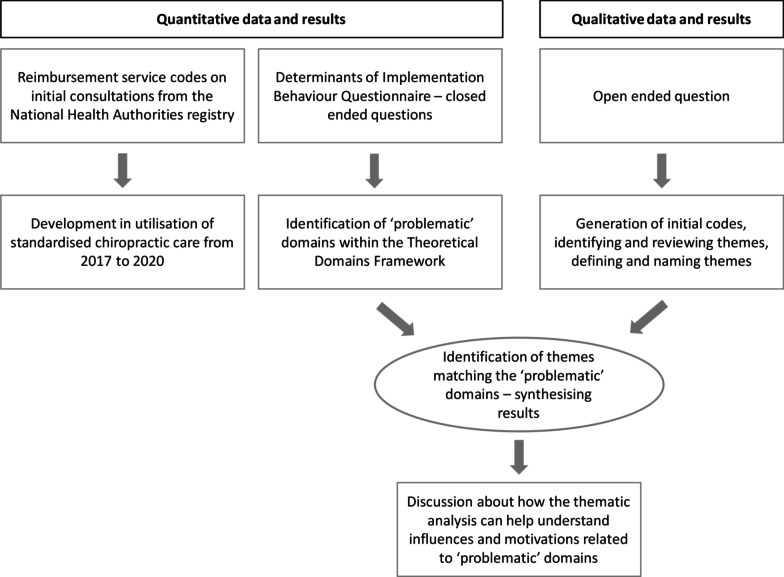
Overall flow of the design

A convergent mixed method design was conceptualized consisting of quantitative and qualitative data collection elements, conducted in parallel [10]. A questionnaire variant of the convergent design was used for this study, including multiple closed-ended and one open-ended questions in a questionnaire. In this design, the qualitative items were an add-on to the quantitative instrument. The rationale for this approach is that qualitative data can provide emergent themes and interesting quotes that can be used to enhance the overall findings and also gain multiple perspectives on the research question [10]. The quantitative data and their subsequent analysis provide a general understanding of the research problem. The qualitative data and their analysis can add to those statistical results by exploring participants’ views beyond the limits of predefined themes of close-ended questions [10] (Fig. 1). The study was reported in accordance with the ‘Good Reporting of A Mixed Methods Study’ (GRAMMS) framework [11].

### Study population and recruitment

To describe the development in the use of standardised chiropractic care packages, clinics receiving reimbursement were identified. Reimbursement provided by the Danish health care system is registered using unique identification numbers related to a company (chiropractic clinic) rather than the chiropractor. Therefore, multiple chiropractors working in the same clinic use the same identification number. In this study, active identification numbers were defined as those with activity (reimbursement) on any chiropractic services described in the collective agreement. Identification numbers were excluded if they were inactivated or established in the data collection period (April 1, 2017, to March 31, 2020).

To explore determinants of potential importance for utilising standardised care packages, all chiropractors working in private clinics in Denmark in 2019 who were members of the Danish Chiropractic Association (DCA) were invited to participate by answering a questionnaire. The DCA estimates that about 95% of all chiropractors in Denmark are members (personal communication).

### Data collection and variables

#### Quantitative data

Service codes on initial consultations were collected on all active identification numbers to explore the overall use of the three care packages. Data were collected per month from April 1, 2017, to March 31, 2020. Danish Regions provided data as part of the National Health Authorities administrating reimbursement to clinics. A detailed description of the standardised care packages is provided in Additional file 1.

Potential determinants of variation in utilisation were assessed using a modified version of the Determinants of Implementation Behaviour Questionnaire (DIBQ) [12]. The original DIBQ questionnaire contains 93 items covering 18 domains. It is based on the ‘Theoretical Domains Framework’ (TDF), a tool developed to apply theoretical approaches to interventions aimed at behaviour change. TDF was built on the synthesis of psychological theories and was validated by Cane et al. [13], who found that it provided a method for the theoretical assessment of implementation processes. The theories are divided into domains describing different areas of potential influence on implementation behaviour.

To tailor the DIBQ for the project’s specific context, the most relevant domains and questions within the domains were selected. The DIBQ questionnaire was reduced to 30 items covering 13 domains considered most relevant for application in the current study. An overview of domains is provided in Table 1, and the single items in the questionnaire are available in Additional file 2. The DIBQ was previously translated into Danish, and feasibility and construct validity was tested using factor analysis [14]. The selection of domains and items used in the current study was done by one of the authors based on previous studies [14–16] and unstructured interviews with chiropractors. Items had to be relevant for the implementation of the standardised care programs and have face validity. All items were answered on a 5-point bipolar Likert scale: (1 = ‘strongly agree’, 2 = ‘agree’, 3 = ‘neutral’, 4 = ‘disagree’ and 5 = ‘strongly disagree’). On April 24, 2019, all Danish chiropractors with a membership of DCA received an e-mail with a link to the modified DIBQ questionnaire using a licensed online survey database ‘SurveyXact’ [17]. The chiropractors had 30 days to respond to the questionnaire. A reminder was sent by e-mail two weeks after the primary invitation.

**Table 1:** Original DIBQ domain included in the modified DIBQ

Domain number	Domains DIBQ	Item numbers*
D1	Knowledge	1–4
D2	Skills	5
D3	Professional role	6–8
D4	Beliefs about capabilities	9–10
D6	Beliefs about consequences	11–16
D7	Intentions	17
D8	Goals	18
D9	Innovation	19–23
D10	Socio-political context	24–25
D12	Patient	26
D13	Innovation strategy	27
D17	Behavioural regulation	28
D18	Nature of the behaviours	29–30

*Items are shown in Additional file 2

The questionnaire had an introduction item examining if the participant had clinical work as a chiropractor in private practice. If the chiropractor had clinical work, information on age, sex, year of education, and country of education was collected, followed by the modified DIBQ. If the chiropractor was not active as a clinician, the questionnaire ended.

#### Qualitative data

For the qualitative part of the study, an open-ended question ‘Do you have further comments regarding the standardised care packages?’ was added at the end of the questionnaire used in the quantitative part of the study. The quantitative and qualitative data were collected from the same respondents, and the study population was the same as described above.

### Analyses and data management

#### Quantitative data

Registry data on the total usage of the initial consultation service codes in all three standardised care packages were calculated for each month over three years. The number of clinics without activity on care packages but otherwise active were calculated per year. The data distribution was assessed, checked for outliers, and presented descriptively as proportions or median and interquartile range (IQR).

For the modified DIBQ baseline, the characteristics of the participating chiropractors were presented as means with standard deviations (SD) and proportions (%). All available data were used to calculate the item responses’ distribution in percentage. The total number of responses on each response option on the 5-point Likert scale (strongly disagree to strongly agree) was calculated within each domain and presented as a percentage. Results were presented graphically, and domains on which more than 20% of the clinicians disagreed or strongly disagreed were identified as ‘problematic’ domains. The 20% cut-point was arbitrarily chosen based on previous suggestions [16] and indications of overall positive attitudes from comparable clinical populations [14]. Within the ‘problematic’ domains, differences between those who agreed or strongly agreed and those who disagreed or strongly disagreed were tested in relation to age, sex, country of graduation, number of chiropractors working in the clinic (1–2 or > 2), and if the clinic had exercise facilities.

Statistical analyses and data management were performed using STATA 16 (Stata Corp, College Station, Texas, USA), and results were graphically displayed using Microsoft Excel 2010 (Microsoft Corporation, Redmond, WA, USA).

#### Qualitative data

A thematic content analysis was conducted [18] by the following steps. All available comments were extracted and displayed in random order. Two assessors (a techno-anthropologist and a chiropractor) independently selected all comments relevant to the topic of inquiry, identified meaning units, and generated initial codes. Themes were searched and reviewed by grouping the codes. The results were compared between the two assessors, and the consensus was reached by discussion. The preliminary results were presented to a consensus group (all co-authors) who had received the results in advance. Adjustments and modifications were discussed, and the results were revised.

#### Merging quantitative and qualitative data

Domains with the most disagreement were selected, and themes relating to the domains were identified. This was done in consensus with two assessors (RKJ and EL). Qualitative findings were then used to expand on the DIBQ data providing further insight into the clinicians’ perspective on standardised care packages.

## Results

### The use of standardised chiropractic care package

**Fig. 2:**
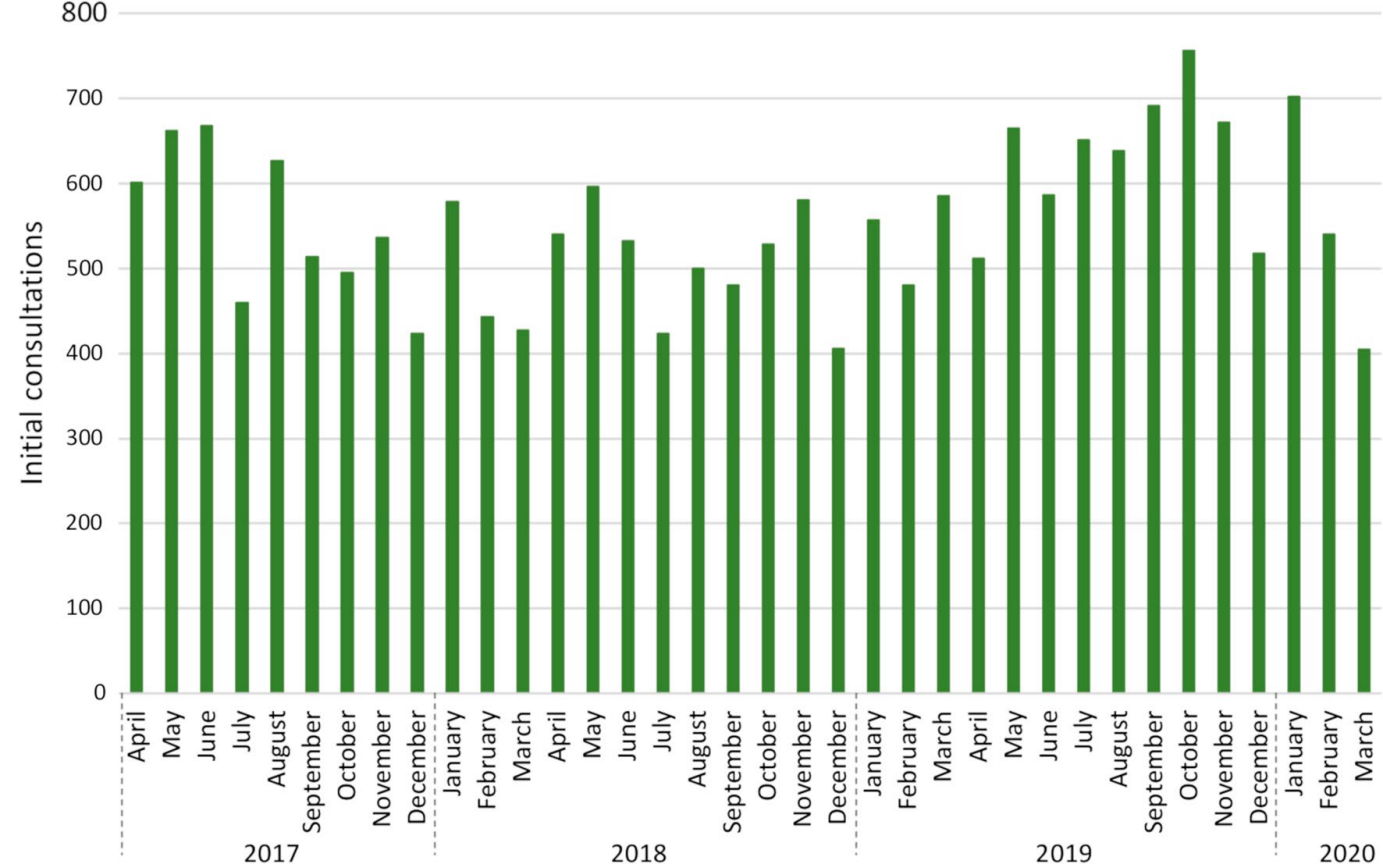
Total activity on initial consultation service codes for standardised care package over a three-year period

**Fig. 3:**
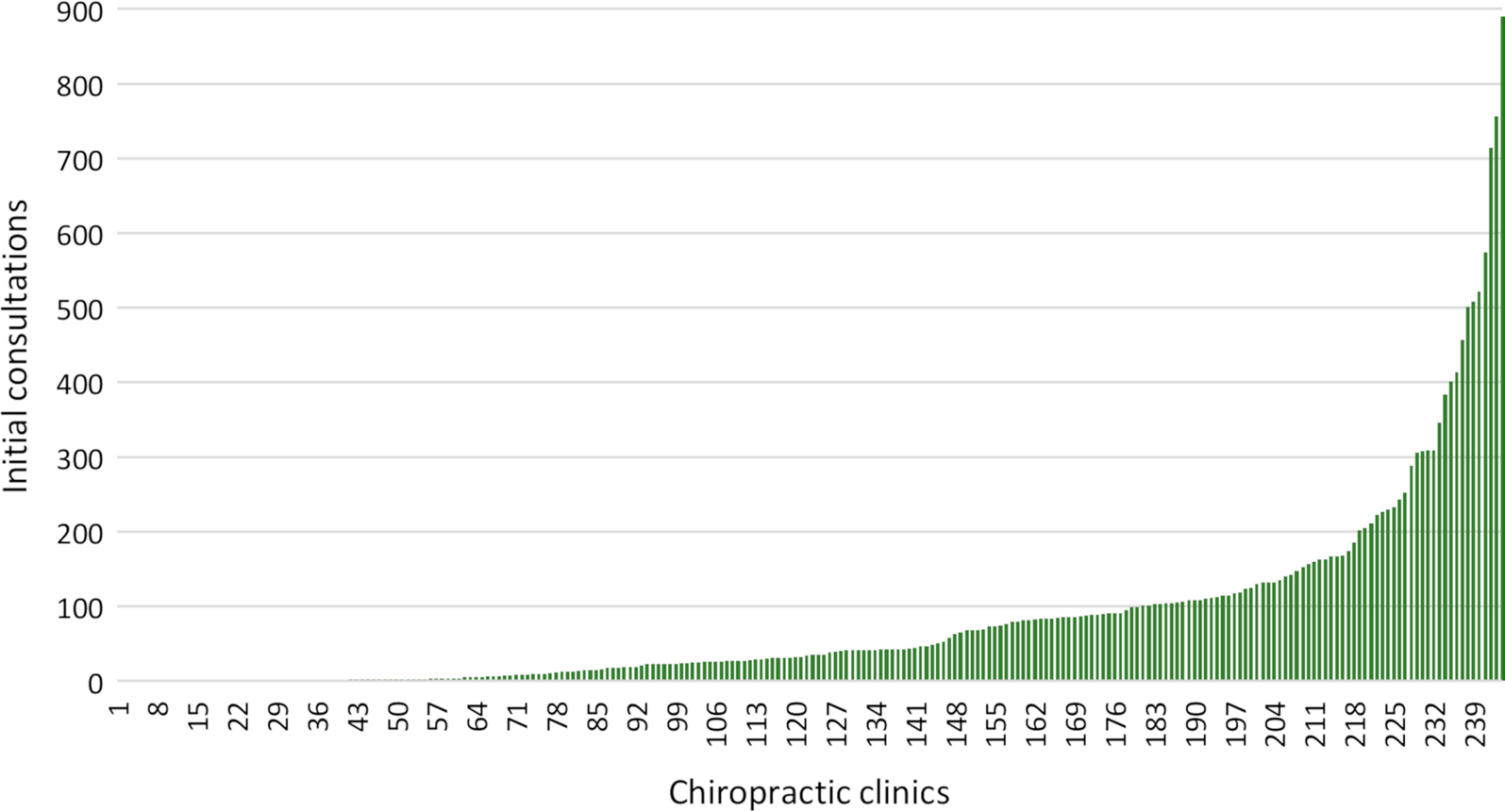
Total activity on initial consultation service codes for standardised care package per clinic (n = 244)

According to the national registration system, 265 unique identification numbers were identified. Of these, 20 were excluded as they were inactive or either inactivated or established in the data collection period. The identification number with the highest registered activity on standardised care package service codes was excluded. It exceeded the second highest activity in the dataset with 389% and was therefore considered an outlier. Finally, a total of 244 active identification numbers receiving reimbursement were included. The total use of service codes on reimbursements for the three care packages’ initial consultations was 19,977. Figure 2 displays total usage per month. The total number of registrations the first year (April 2017 to March 2018) was 6,434, the second year (March 2018 to April 2019) 6,207, and the third year (April 2019 to March 2020) 7,336. In the first year, 63 (26%) of the 244 active identification numbers did not have any registered service codes on standardised care packages, the second year, it was 55 (23%), the third year 45 (18%), and 32 (13%) did not have any registrations in all three years. Sixty-one clinics (25%) had less than five registrations. The highest number of registrations per month for a clinic was 32, and the median activity per month for all clinics was 0.96 (IQR 0–5.7). Of the 244 clinics, 67 (26%) had 100 or more service codes, and together, they accounted for 75% of the total activity. Sixteen clinics (11%) were responsible for 50% of the total activity (Fig. 3).

### Modified DIBQ

**Fig. 4:**
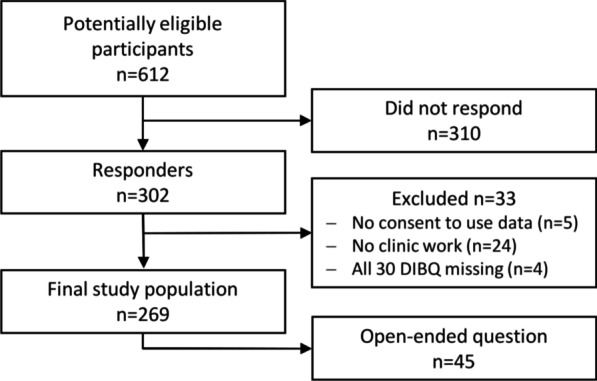
Flowchart of data collection

The modified DIBQ was sent to 612 chiropractors, and 302 (49%) responded. Of the 302 chiropractors, 33 were excluded: 5 did not provide written consent to use data, 24 did not have clinical work, and 4 did not answer any DIBQ questions. The final study population consisted of 269 (44%) chiropractors (Fig. 4).

The mean age of the included respondents was 45 (SD 11.3) and 58.5% were women. Fifty-five respondents provided a comment to the open-ended question, of which 45 were related to the standardised care packages and included in the qualitative analysis. Characteristics of the study populations are shown in Table 2. There were no statistically significant differences in baseline characteristics in those who provided a comment in the open-ended question for the qualitative analysis and those who did not.

**Table 2:** Characteristics of chiropractors answering DIBQ and the subsample answering the open-ended question

	DIBQ (n = 269)	Open-ended question (n = 45)
Age	n = 229	n = 37
Mean (SD)	45 (11.3)	42.9 (10.2)
Range	25–69	27–67
Sex, n (%)	n = 268	n = 45
Female	157 (58.5)	26 (57.8)
Year of graduation, n (%)	n = 261	n = 42
1970–1979	13 (5.0)	3 (7.1)
1980–1989	48 (18.4)	3 (7.1)
1990–1999	48 (18.4)	7 (16.7)
2000–2009	81 (31.0)	17 (40.5)
> 2010	71 (27.2)	12 (28.6)
Country of graduation, n (%)	n = 269	n = 45
Denmark	152 (56.5)	30 (66.7)
UK	58 (21.6)	8 (17.8)
USA	55 (20.5)	7 (15.6)
Canada	4 (1.5)	0 (0.0)
Number of chiro’s working in the clinic, n (%)	n = 264	n = 43
1	42 (15.9)	5 (11.6)
2–3	85 (32.2)	20 (46.5)
4–5	91 (34.5)	11 (25.6)
6–7	33 (12.5)	5 (11.6)
> 7	13 (4.9)	2 (4.7)
Exercise facilities in the clinic, n (%)	n = 268	n = 45
Yes	107 (40)	18 (40.0)
No	161 (60)	27 (60.0)
Partially reimbursement agreement, n (%)	n = 267	n = 45
Yes	259 (97)	45 (100.0)
No	8 (3)	0 (0.0)

Of the 269 answering the modified DIBQ, 88.9% had no missing items, 5.2% had one missing, 1.9% had 2–10 items missing, 2.6% had 11–20 items missing, and 1.5% had 21–24 items missing.

In general, the responses indicated a positive attitude towards utilising the standardised care programs as at least 50% of the clinicians answered ‘Strongly agree’ or ‘Agree’ to 27 out of the 30 items (Additional file 2).

**Fig. 5:**
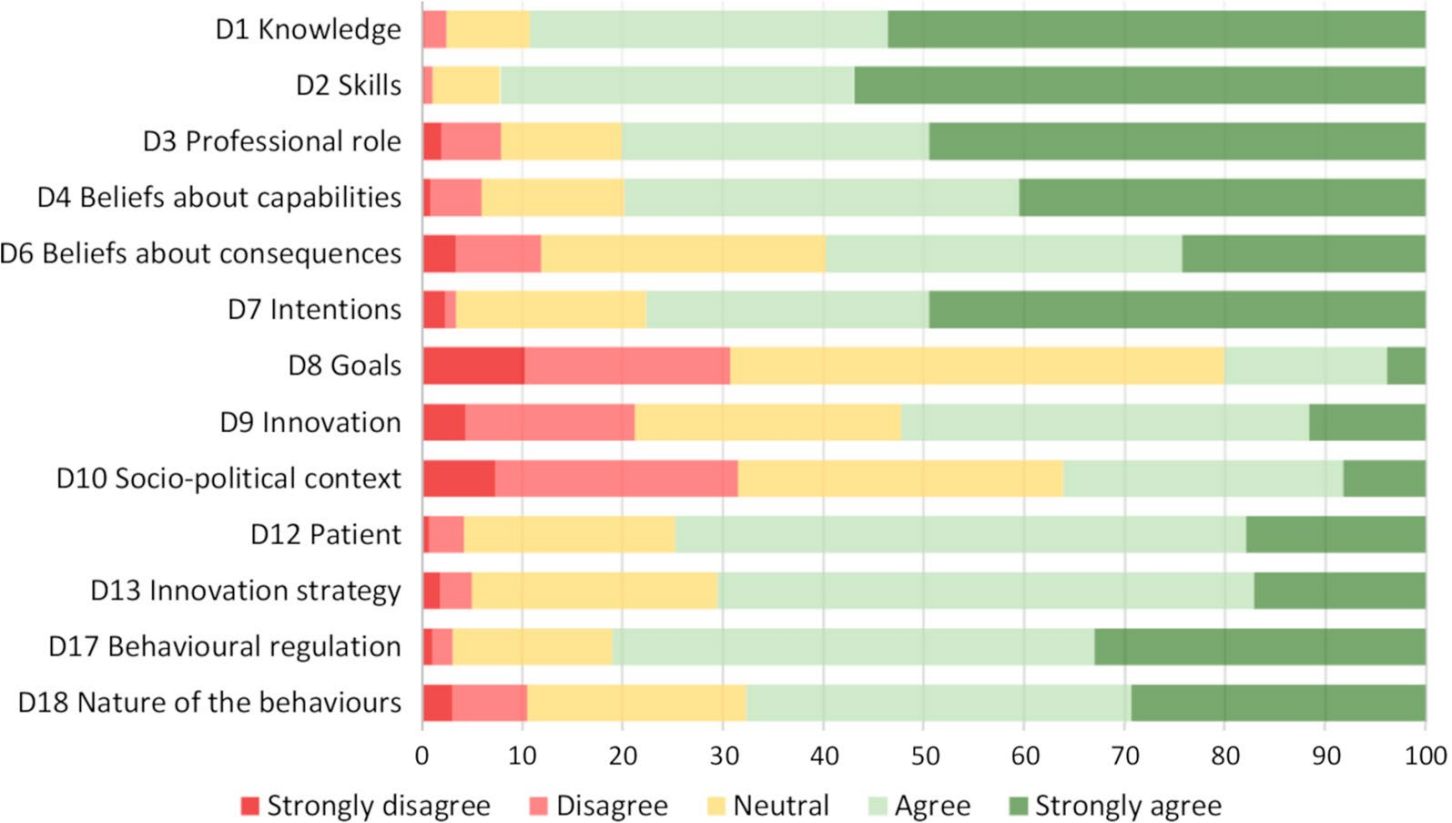
Distribution of responses within domains

Figure 5 illustrates the distribution of responses within the 13 domains. In 10 domains, the overall agreement was highly reflecting a positive attitude. Three domains showed a different pattern and were identified as ‘problematic’ domains. Problematic domains were domains where more than 20% of clinicians either disagreed or strongly disagreed. In domain ‘D8 Goals’ (1 item), 31% disagreed or strongly disagreed that delivering the standardised care packages was a high priority. These clinicians were older (mean age 48.7 (SD 11.7)) compared to those who agreed or strongly agreed (mean age 41.76 (SD 11.0), p = 0.002), and they less often had exercise facilities in the clinic (50% versus 68%, p = 0.04).

In domain ‘D9 Innovation’ (5 items), 21% of the clinicians disagreed or strongly disagreed. This group was a little older (mean age 46.8 (SD 11.1) compared to those who agreed or strongly agreed (mean age 43.8 (SD 10.8), p < 0.001), were more often educated in the USA or UK (50%) compared to those who agreed or strongly agreed (38%) and had less often exercise facilities in the clinic (56% versus 68%, p = 0.001). Disagreement was related to statements claiming that standardised care packages took little time and were simple to deliver, were compatible with daily practice, and that it was possible to tailor them to patients’ and chiropractors’ needs.

In the domain ‘D10 Socio-political context’ (2 items), 32% of the clinicians disagreed. Disagreement was primarily driven by one item asking if ‘Primary Health Care’ was sufficiently oriented towards delivering the standardised care packages. No differences between those who agreed and disagreed were found.

### Clinicians’ perspectives

Categorisation and analysis of the qualitative data led to the emergence of 4 themes described below: ‘A clash with the organization of clinical practice’, ‘The chiropractor’s role’, ‘No usage of care packages’ and ‘Positive attitudes and new ideas’. Key quotes are presented in Table 3.

**Table 3:** Joint display of quantitative and qualitative results

Quantitative results	Qualitative results	Key quotes supporting the themes	ID
Domains	Themes		
D8 goals	No usage of care packages	The standardised care package for lumbar spinal stenosis is less integrated into the workflow in the clinic. There are fewer patients, and there are often other care providers involved	2
		My primary patient group is children and chronic pain patients, and in those two categories, I rarely find anyone who can be included in the standardised care packages	6
		I see a lot of babies at the clinic, and therefore I probably have a low number of standardised care packages statistically, but it is not because I cannot or will not use the care packages	35
	A clash with the organization of clinical practice	However, the many established time-fixed dates take focus away from the treatment of patients and are more time-consuming in general	52
		No two patients are alike—there is a need for individual adaptations in the care pathway	19
	The chiropractor’s role	Could be nice with a better definition of the chiropractor’s role concerning the spinal stenosis care package. What is expected of a treatment course, what are the treatment options, etc.?	3
		The care package for spinal stenosis is still a mystery! Yes, to the exercise program, information/advice, and treatment—but it is a chronic condition that will not be cured after three months—unless one makes sense and sends them for surgical evaluation	14
D9 innovation	A clash with the organization of clinical practice	I choose to inform [the GP] when relevant e.g. if there is a need for painkillers, sick leave, referral for physiotherapy, or if I have referred the patient for MRI	10
		And holidays, days off, and patients’ who don’t turn up make it practically silly with the time-fixed follow-ups. […] Overall, our standardised care packages are very impractical and have little to do with sound reality. In my opinion, a neurological examination should always take place in case of worsening—whether it is 2, 4, or 8 weeks after starting. […] Maybe time-fixed neurological examinations can result in doing examinations ‘heedlessly’?	14
		No more care packages should be designed for other types of patients. It will end in a chaos of codes, deadlines, etc. It’s annoying to have to keep an eye on whether it’s time for this or that all the time	20
		I find it a little difficult to remember 2, 4, and 8 weeks of follow-up—as at each and every treatment, I ask the patients about symptoms and perform tests when I feel there is a need. I also work on remembering to register the different service codes at the 2, 4, and 8 weeks of follow-up, but often forget it	28
		Can be a good mechanism for not forgetting to re-evaluate continuously for some people but in terms of my way of practising it is odd, as testing, re-testing, and ongoing re-evaluation, etc. are part of my daily routine	49
D10 socio-political context	A clash with the organization of clinical practice	GPs often encourage the patient to withdraw from the standardised care package and see a physiotherapist instead	23
		Letters [to GPs] have not yet resulted in a single answer or response from any GP	14
	The chiropractor’s role	My challenge in using standardised care packages is to make them visible to patients. I live in [xxx] part of the country, and in this area, the GPs do not think we [chiropractors] should have the competencies and coordinate care packages concerning patients with disc herniations. Therefore, it is difficult to enrol patients unless they see me before their GP. It’s an uphill task	15
		I would appreciate it if the monitor part was with the GPs and the manual part with us [chiropractors]. The reality is that when I enrol a patient in a standardised care package, I have all the responsibilities. I do not want that—to be completely honest. I want to be the biomechanical coordinator, and as previously, let the GP take care of the social challenges and medical pain treatment	46
		[…] it can be difficult to explain to the patient the indication for a further charge at certain time-fixed consultations as my routine has not changed significantly	49

#### A clash with the organization of clinical practice

Several aspects of clinical practice were challenged by implementing standardised care packages. The structure of the standardised care packages collided with the usual organization of clinical practice that clinicians regarded more effective and efficient. The logistics did not comply with the diversity in the clinical course of patients and therefore did not comply with reality. There was no room for adapting the care package structure if the clinical course of the patient did not fit the predefined logistics (ID: 14, 19). The time-fixed follow-up sessions were difficult to comply with, and it was hard to remember to book patients at the right time (ID: 14, 28). The focus on logistics took attention away from more important tasks such as patient treatment (ID: 52). In patients with radiculopathy, clinicians would usually examine patients systematically at each consultation or at indication, and the sudden focus on time-fixed examinations did not fit their usual clinical practice (ID: 49). Also, there were concerns that it could devalue existing clinical standards (ID: 14). Standardised communication with the general practitioner (GP) was not in line with usual clinical practice, did not improve clinical standards, and was time-consuming (ID: 10, 14, 52). Finally, the economic structure did not fit clinical practice. When clinicians delivered more or less the same content at every consultation, it was hard to explain to patients why they had to pay more at specific consultations (2, 4, and 8 weeks) (ID: 49).

#### No usage of care packages

One of the main reasons for not using standardised care packages was that clinics had specialised in treating children. Therefore, they did not see a lot of other patients, including those with radiculopathy or spinal stenosis (ID: 6, 35).

#### The chiropractor’s role

Chiropractors in Denmark have a university-grounded five-year chiropractic degree and tight integration with the National Health Care system. Therefore, the professional responsibility has steadily increased and now includes managing all parts of a treatment course in patients with musculoskeletal problems. However, it is possible that not all chiropractors feel comfortable in this role. Also, some experienced resistance from local GPs that made it hard to enrol patients in a clinical care package with the chiropractor as coordinator (ID: 3, 15, 46).

#### Positive attitudes and new ideas

In general, there were positive attitudes towards standardised care programs. It was regarded as a supportive tool in the management of more complicated or severe diagnoses, and although it took a while to get implemented, it was worthwhile. New ideas included suggestions on improving the existing care packages by more significant reimbursement, digital help functions to remember time-fixed follow-ups, increased information to GPs about the existence of care packages, and suggestions about packages aimed at other patient groups.

### Linking data analysis from the quantitative and qualitative design

As described above in the quantitative results section, three domains were identified as problematic (D8 Goals, D9 Innovation, and D10 Socio-political context). Themes (supported by key quotes) expanding insight to the ‘problematic’ domains were identified. Table 3 shows a joint display of how the qualitative themes supported by quotes provide a more complete understanding of the three domains.

## Discussion

Of the 244 included clinics, the total use of initial consultations in the three care packages was relatively stable over time. However, the use was limited and inconsistent as 32 clinics (13%) did not have any registrations across all three years, and 16 clinics (11%) were responsible for 50% of the total activity. Danish chiropractors were generally positive towards the standardised care packages as at least 60% either ‘agreed’ or ‘strongly agreed’ in 10 out the 13 domains on DIBQ. Three ‘problematic’ domains were identified. They included ‘Socio-political context’ with 32% of the clinicians’ disagreeing that primary health care was sufficiently oriented towards the delivery of the standardised care packages, ‘Goals’ with 31% disagreeing that delivering the standardised care packages was a high priority, and ‘Innovation’ with 21% disagreeing that the standardised care packages took little time and were simple to deliver, and that it was possible to tailor them to patients’ and chiropractors’ needs. Four themes from the qualitative data were identified, of which one ‘Positive attitudes and new ideas’ supported the overall positive answers in the DIBQ. Three themes, ‘A clash with the organization of clinical practice’, ‘No usage of care packages’ and ‘The chiropractor’s role’ expanded insight to the ‘problematic’ domains as they provided a more complete understanding of the three domains.

The variation in the use of standardised care packages could be explained by the diversity in patient groups that clinics attract. A previous study on website claims from Danish chiropractors showed that 80% of websites mention infants and children as special interest groups, and almost 60% focus on athletes [19]. In comparison, only 12% of the websites mentioned the standardised care packages for radiculopathy and 4% for lumbar spinal stenosis (unpublished data). This was supported by quotes explaining that some clinics primarily see children and that management of lumbar spinal stenosis was less integrated (ID: 2, 6, 35). However, the absence of specific patient groups only seems to be part of the explanation. Also, differences in the number of chiropractors in a clinic could influence the use of care packages. Larger clinics with many chiropractors will naturally have a higher activity than smaller clinics, although it does not explain the large proportion of clinics with only few or no registered care packages.

Another issue that could have influenced the use of care packages was other collaborators in the nearby health care system. One-third of the clinicians disagreed that primary health care was sufficiently oriented towards delivering the standardised care package. This was elaborated on by quotes explaining that they experienced resistance from GPs to cooperate on providing the care packages and that some GPs even counteracted the chiropractor (ID: 15, 23). Written communication informing GPs about patient status is a mandatory part of the care packages described in the collective agreement. However, for a chiropractor whose income depends on patient flow, it is contra-intuitive to systematically inform GPs if the clinician suspects that the GP will likely advise the patient to discontinue the standardised care program. Though, clinicians could also be biased towards GPs. Results from a previous survey among chiropractors suggested that the majority did not consider GPs as active collaborators in their interprofessional service delivery [20].

The extra reimbursement applied to the standardised care packages resulted in fewer expenses for the patient (Additional file 1). Therefore, the chiropractors did not experience a direct extra income for the extra administration tasks associated with the care packages. The incentive for chiropractors is more likely associated with lower prices being affordable for more patients and thereby warrant a larger patient flow. The same incentive could apply to chiropractors attracting more patients as they enjoy a reputation as an evidence-based profession that integrates with the national health system proven by the willingness to provide larger reimbursement. However, it is unclear if the indirect incentive is strong enough to ensure usage or if it is sufficiently understood by clinicians. Although the most are positive about taking on a more professional role, some do not want the extra responsibility (ID: 46) or do not feel well prepared for the task (ID: 3). It may be favourably for future implementation initiatives to focus on these issues and embrace the groups that are not ready for the task.

The theme ‘A clash with the organization of clinical practice’ explained many of the issues in the domain ‘Innovation’ where clinicians disagreed that the standardised care packages took little time and were simple to deliver and that it was possible to tailor them to patients’ and chiropractors’ needs. Clinicians found the structure of the care programs time-consuming and inflexible. Especially the time-fixed follow-ups challenged usual practice by providing artificial logistics that did not fit the relatively large variation in patients’ symptoms and demands. This is in line with the findings of a scoping review from Sorondo et al. [21], showing that two of the five main barriers to clinical guideline utilisation were that guidelines were not specific to individual patients and that they were time-consuming.

In the present study, there were concerns that the structure of the standardised care packages resulted in a downgrading of the services the clinicians usually provided to this type of patient. Also, the preplanned logistics that required testing at specific time points could create a false sense of security that would reduce clinical reasoning (ID: 14). However, chiropractors also saw the structure as an advantage and suggested that it provided security to patients. Further exploration of both positive and negative experiences could provide a basis for knowledge sharing for both clinicians and stakeholders.

### Clinical implications and future research

The variety in the use of the standardised care packages could result in differences in what chiropractors offer patients with radiculopathy or lumbar spinal stenosis. Although it is possible within the collective agreement to offer the same chiropractic services (the same content) as in the standardised care packages, there would likely be a difference in payment for the patient. Also, the variation could be a potential threat to the confidence the profession enjoys from the national health care system as it is unlikely that a variation of this size was related to natural variation in disease prevalence or special interest (e.g., children).

Until now, the implementation of the standardised care packages has focused on increasing awareness of the content and administration of the collective agreement contracted in 2017. The DCA has initiated information meetings in each of the five regions of Denmark, described the content in a national professional journal for chiropractors distributed to all DCA members, and in e-mails with newsletters including descriptions and links. Also, the DCA has provided booklets aimed at both clinicians and patients with an overview of the content and standardised text to use in the communication with the patients’ GP. In 2018, further initiatives were implemented, such as a podcast about getting started and personal contact to the clinics with very low or no use of the standardised care packages [6]. To further explore implementation facilitators and barriers, it would be beneficial to conduct a large-scale qualitative study based on the results of the present study.

### Strengths and limitations

The registration codes on initial consultations provided by Danish Regions are complete and comprehensive. However, the registry only records information from chiropractic clinics with a reimbursement agreement which applies to 86% of all clinics in Denmark, according to a survey from 2014.

The 30 DIBQ items were selected by only one researcher based on a previous translation and selection process [14], face validity, and unstructured interviews with chiropractors. It is possible that a more structured item selection process with discussion boards and user involvement would have included other domains and provided diverse results. However, as the emerging themes fitted well with DIBQ results, it seems likely that the most relevant domains were covered.

The response rate on DIBQ was 49% which could introduce selection bias. This response is lower than previous surveys ranging from 75 to 82% [22], although the characteristics of the respondents are comparable on age, sex, country of education, and year of graduation. However, this could challenge the external validity as it is possible that the very positive responses on most items do not reflect the other 51% of the profession and that other barriers would emerge if a larger proportion was included. Therefore, further research on the topic is necessary, and future qualitative studies should also aim to sample from the non-responder group.

Only 3% of the chiropractors who responded to the questionnaire in 2019 worked in clinics that did not receive partial reimbursement. In comparison, unpublished survey data show that the proportion of clinics not receiving reimbursement in 2018 was 11% and 4% in 2020 [23]. Although data are not directly comparable, this subpopulation is likely underrepresented in our sample. We assume that chiropractors working in clinics without reimbursement will be less likely to use the care packages and, therefore, less likely to answer the questionnaire. However, they would also be less qualified to answer questions concerning the implications of care packages. The use of care packages by the respondents was not directly addressed. The single open-ended question included for the qualitative component was not anchored to any of the domains investigated and was only answered if the respondent chose to. Although there was no opportunity to probe for further response in the questionnaire and the open-ended question was answered by only 45 participants, it provided enough detail to conduct a preliminary exploration of themes relevant to the profession but does not provide sufficient quality to obtain a deeper understanding. To fully explore barriers and facilitators to the utilisation of standardised care packages, an in-depth qualitative study is needed.

## Conclusion

Based on responses from half of the Danish chiropractors, the profession generally appeared positive towards standardised care packages, although there was a considerable variation in use between clinics. Low utility seemed mainly related to logistics and organization of standardised care packages, the chiropractor’s role, collaboration with GPs, and the patient population of interest to the clinic.

## Supplementary Information

**Additional file 1:** A detailed description of the standardised chiropractic care packages.

**Additional file 2:** Items of DIBQ questionnaire, related domains, and distribution of responses to individual items.

## Data Availability

The datasets used and/or analysed during the current study are available from the corresponding author on reasonable request.

## Abbreviations

DCA: Danish chiropractic association
DIBQ: Determinants of implementation behaviour questionnaire
GP: General practitioner
GRAMMS: Good reporting of a mixed methods study
IQR: Interquartile range
SD: Standard deviations
TDF: Theoretical domains framework

## References

[1] Gillam S, Siriwardena AN (2014). Regulation in primary care. Qual Prim Care.

[2] National Agreement on Chiropractic 2021 (in Danish). https://www.danskkiropraktorforening.dk/media/2279/kiro_ok21_web.pdf. Accessed 24 Aug 2021.

[3] Kjaer P, Kongsted A, Hartvigsen J, Isenberg-Jorgensen A, Schiottz-Christensen B, Soborg B, et al. National clinical guidelines for non-surgical treatment of patients with recent onset neck pain or cervical radiculopathy. Eur Spine J. 2017. 10.1007/s00586-017-5121-8 28523381

[4] Stochkendahl MJ, Kjaer P, Hartvigsen J, Kongsted A, Aaboe J, Andersen M (2018). National Clinical Guidelines for non-surgical treatment of patients with recent onset low back pain or lumbar radiculopathy. Eur Spine J.

[5] Rousing R, Jensen RK, Fruensgaard S, Strom J, Brogger HA, Degn JDM (2019). Danish national clinical guidelines for surgical and nonsurgical treatment of patients with lumbar spinal stenosis. Eur Spine J.

[6] Danish Chiropractic Association (in Danish). https://www.danskkiropraktorforening.dk/nyheder/ok-statistik-for-hele-2018/. Accessed 24 Aug 2021.

[7] National Agreement on Chiropractic 2017 (in Danish). https://www.danskkiropraktorforening.dk/media/1122/55501-kiropraktik-ny-ok-opdateret-19-april-17.pdf. Accessed 24 Aug 2021.

[8] Jensen RK, Harhangi BS, Huygen F, Koes B (2021). Lumbar spinal stenosis. BMJ.

[9] Jensen RK, Kongsted A, Kjaer P, Koes B (2019). Diagnosis and treatment of sciatica. BMJ.

[10] Creswell JW, Clark VLP (2018). Designing and conducting mixed methods research.

[11] O'Cathain A, Murphy E, Nicholl J (2008). The quality of mixed methods studies in health services research. J Health Serv Res Policy.

[12] Huijg JM, Gebhardt WA, Dusseldorp E, Verheijden MW, van der Zouwe N, Middelkoop BJ (2014). Measuring determinants of implementation behavior: psychometric properties of a questionnaire based on the theoretical domains framework. Implement Sci.

[13] Cane J, O'Connor D, Michie S (2012). Validation of the theoretical domains framework for use in behaviour change and implementation research. Implement Sci.

[14] Ris I, Schroder K, Kongsted A, Abbott A, Nilsen P, Hartvigsen J (2021). Adapting the determinants of implementation behavior questionnaire to evaluate implementation of a structured low back pain programme using mixed-methods. Health Sci Rep.

[15] Ris I, Boyle E, Myburgh C, Hartvigsen J, Thomassen L, Kongsted A. Factors influencing implementation of the GLA: D Back, an educational/exercise intervention for low back pain: a mixed-methods study. JBI Evid Implement. 2021. 10.1097/XEB.0000000000000284 PMC8635265 33965996

[16] Schroder K, Oberg B, Enthoven P, Kongsted A, Abbott A (2020). Confidence, attitudes, beliefs and determinants of implementation behaviours among physiotherapists towards clinical management of low back pain before and after implementation of the BetterBack model of care. BMC Health Serv Res.

[17] SurveyXact. www.surveyxact.dk. Accessed Jun 2020.

[18] Anderson R. Thematic content analysis (TCA): descriptive presentation of qualitative data 2007.

[19] Jensen RK, Agersted MEI, Nielsen HA, O'Neill S (2020). A cross-sectional study of website claims related to diagnoses and treatment of non-musculoskeletal conditions. Chiropr Man Therap.

[20] Myburgh C, Christensen HW, Fogh-Schultz AL (2014). Chiropractor perceptions and practices regarding interprofessional service delivery in the Danish primary care context. J Interprof Care.

[21] Sorondo D, Delpierre C, Cote P, Salmi LR, Cedraschi C, Taylor-Vaisey A (2021). Determinants of clinical practice guidelines' utilization for the management of musculoskeletal disorders: a scoping review. BMC Musculoskelet Disord.

[22] Nielsen OL, Kongsted A, Christensen HW (2015). The chiropractic profession in Denmark 2010–2014: a descriptive report. Chiropr Man Therap.

[23] Chiropractic Knowledge Hub. KiroFAKTA (in Danish). https://kiroviden.dk/forskning/afhandlinger-og-rapporter. Accessed 12 Jan 2022.

[24] The Danish National Committee on Health Research Ethics. Act on research ethics review of health research projects (Updated March 18 2021). https://en.nvk.dk/. Accessed 15 Sept 2021.

