# Supplementary material for: The utilisation of regulated standardised care packages by Danish chiropractors: a mixed methods study
Source: Chiropr Man Therap. 2022 Mar 8;30:14. doi: 10.1186/s12998-022-00423-7 (PMC8903550; doi:10.1186/s12998-022-00423-7)
Supplement: Supplementary file 1 — Additional file 1: A detailed description of the standardised chiropractic care packages. [file 12998_2022_423_MOESM1_ESM.pdf]

Additional file 1

A detailed description of the standardised chiropractic care packages

In Denmark, chiropractors are regulated by the Danish National Health Authorities. The terms of regulation including partial reimbursement are negotiated every fourth year between Danish Health Authorities and the Danish Chiropractic Association (DCA). This agreement includes three standardised chiropractic care packages for lumbar spinal stenosis, lumbar radiculopathy, and cervical radiculopathy, respectively [1].

The standardised care packages describe a management structure and logistics of the patients' care pathway that the chiropractors are obligated to comply with. Management includes time-fixed follow-up sessions at two, four, and eight weeks from baseline to monitor the progression of symptoms in the case of lumbar or cervical radiculopathy (Figure 1) and four and twelve weeks in case of lumbar spinal stenosis. A consultation includes case history and clinical examination related to the specific diagnosis, reassessment of the treatment plan based on the status of the patient, medical record-keeping, and standardised written communication with the patient's general practitioner (GP). Treatment modalities (information, exercise, etc.) are not described in detail in the agreement which instead refers to the recommendations of current national clinical guidelines [2-4].

Examples of fees and reimbursement are described in Figures 1-3.

Figure 1 describes the logistics of the standardised care program for lumbar or cervical radiculopathy with time-fixed follow-up consultations (red bars) and 'as needed' consultations (blue areas) within a fixed time frame. The program for lumbar spinal stenosis is similar except for only two time-fixed follow-up sessions at four and twelve weeks, respectively.

Figure 1 Standardised care program for patients with cervical or lumbar radiculopathy illustrating fixed and 'as needed' consultations

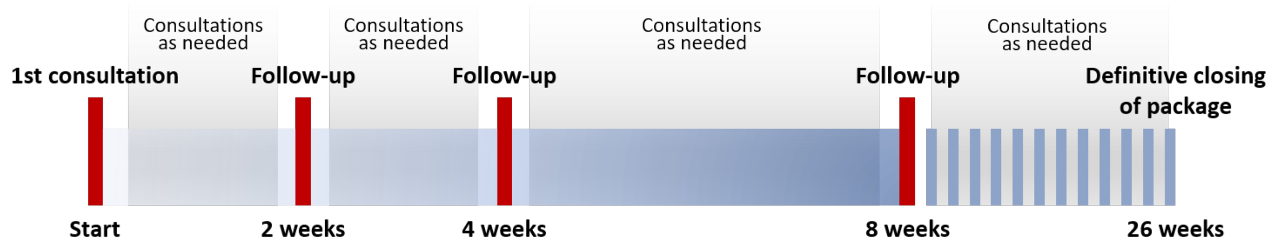

The red bars illustrate time-fixed mandatory consultations and the blue areas illustrate the time frame of which consultations 'as needed' can be utilised.

Figure 2 illustrates the differences in price per consultation, the reimbursement, and the final patient price for an average standardised care program and a 'normal' care pathway. The prices (in EUR) correspond to the codes from the collective agreement from April 2021 [1]. Price codes named 104x refers to lumbar radiculopathy, 105x to cervical radiculopathy, and 106x to lumbar spinal stenosis. For the standardised care package, the 1st consultation is code 1040, 1050, or 1060, time-fixed follow-up consultations are code 1042-44, 1052-54, or 1062-63 and consultations 'as needed' are code 1045, 1055, or 1065. For the 'normal' care program the 1st consultation is code 1015, and consultations, as needed, are calculated as an average of 1035, 1036 and 1037, because they vary in price depending on the services provided.

Figure 2: Standardarised care program for patients with cervical or lumbar radiculopathy illustrating fixed and 'as needed' consultations

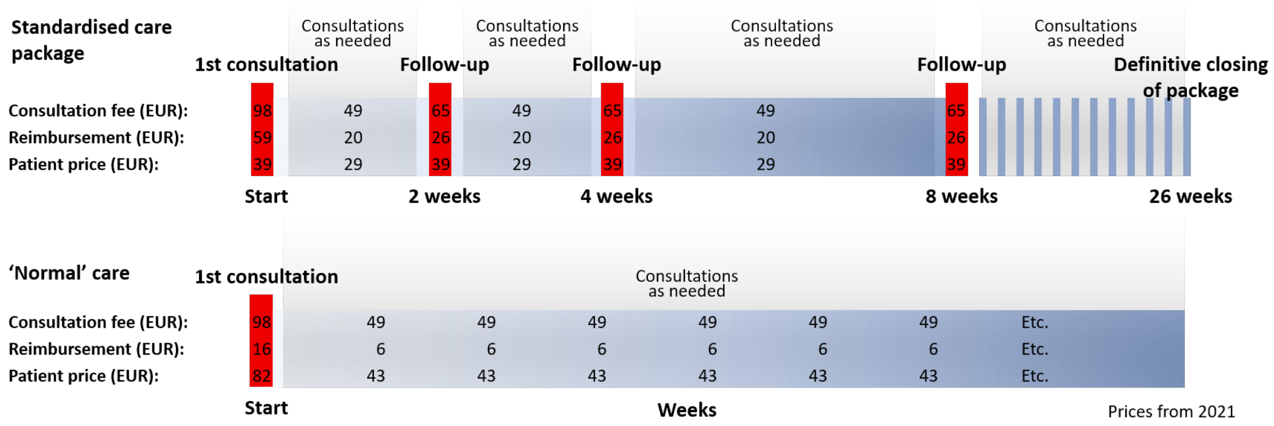

Figure 3 illustrates the full price of a standardised care package with 10 consultations compared to a 'normal' care pathway with 10 consultations. The prices (in EUR) correspond to the rates from the collective agreement from April 2021 [1]. Corresponding price codes are described above.

Figure 3: Standardarised care program for patients with cervical or lumbar radiculopathy illustrating fixed and 'as needed' consultations in an example with 10 consultations

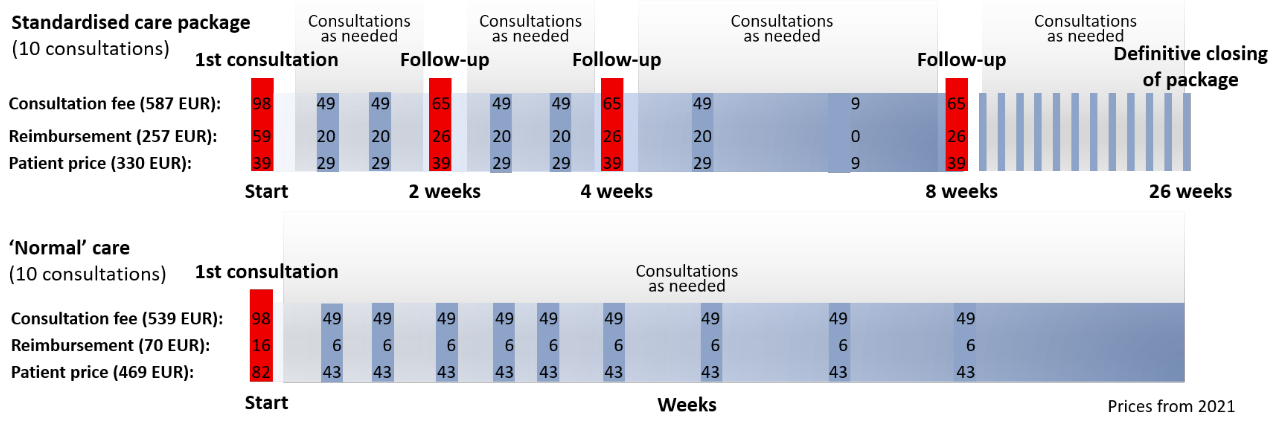

Reference list

1. National Agreement on Chiropractic 2021 [in Danish]. https://www.danskkiropraktorforening.dk/media/2279/kiro_ok21_web.pdf. Accessed 24 August 2021

2. Rousing R, Jensen RK, Fruensgaard S, Strom J, Brogger HA, Degn JDM, et al. Danish national clinical guidelines for surgical and nonsurgical treatment of patients with lumbar spinal stenosis. Eur Spine J. 2019;28(6):1386-96.

3. Stochkendahl MJ, Kjaer P, Hartvigsen J, Kongsted A, Aaboe J, Andersen M, et al. National Clinical Guidelines for non-surgical treatment of patients with recent onset low back pain or lumbar radiculopathy. Eur Spine J. 2018;27(1):60-75.

4. Kjaer P, Kongsted A, Hartvigsen J, Isenberg-Jorgensen A, Schiottz-Christensen B, Soborg B, et al. National clinical guidelines for non-surgical treatment of patients with recent onset neck pain or cervical radiculopathy. Eur Spine J. 2017.
