# Supplementary material for: The utilisation of regulated standardised care packages by Danish chiropractors: a mixed methods study
Source: Chiropr Man Therap. 2022 Mar 8;30:14. doi: 10.1186/s12998-022-00423-7 (PMC8903550; doi:10.1186/s12998-022-00423-7)

Additional file 2

Items of DIBQ questionnaire, related domains, and distribution of responses to individual items

Items, original DIBQ domain, and construct included in the modified DIBQ

Domain number	Domains DIBQ	Item number	Constructs	Items
D1	Knowledge	1	Knowledge	I know how to deliver standardised care packages.
		2	Role clarity	Objectives of the standardised care packages and my role in this are clearly defined for me.
		3	Role clarity	With regard to the standardised care packages, I know what my responsibilities are.
		4	Role clarity	In my work with the standardised care packages, I know exactly what is expected from me.
D2	Skills	5	Skills	I have the skills to deliver the standardised care packages.
D3	Professional role	6	Professional role	Delivering the standardised care packages is part of my work as a chiropractor.
		7	Professional role	As a chiropractor, it is my job to deliver standardised care packages.
		8	Professional role	It is my responsibility as a chiropractor to deliver the standardised care packages.
D4	Beliefs about capabilities	9	Self-efficacy	I am confident that I can deliver the standardised care packages.
		10	Self-efficacy	I am confident that I can deliver the standardised care packages even when there is little time.
D6	Beliefs about consequences	11	Attitude	For me, delivering the standardised care packages is (not useful at all – very useful).
		12	Attitude	For me, delivering the standardised care packages is (not worthwhile at all – very worthwhile).
		13	Outcome expectancies	If I deliver the standardised care packages according to the collective agreement, the standardised care packages will be most effective.
		14	Outcome expectancies	If I deliver the standardised care packages according to the collective agreement, participants will appreciate this.

		15	Outcome expectancies	If I deliver the standardised care packages according to the collective agreement, this will strengthen the collaboration with the general practitioners.
		16	Outcome expectancies	If I deliver the standardised care packages according to the collective agreement, it will help participants to be able to cope better with their back problems.
D7	Intentions	17	Intensions	I will definitely deliver the standardised care packages in the next three months.
D8	Goals	18	Priority	How often is working on something else on your agenda more urgent than delivering the standardised care packages? [Rephrased to fit answering options: *There is rarely something else on your agenda more urgent than delivering the standardised care packages*]
D9	Innovation	19	Innovation characteristics	It is possible to tailor the standardised care packages to participants' needs.
		20	Innovation characteristics	It is possible to tailor the standardised care packages to the chiropractors' needs.
		21	Innovation characteristics	The standardised care packages cost little time to deliver.
		22	Innovation characteristics	The standardised care packages are compatible with daily practice.
		23	Innovation characteristics	The standardised care packages are simple to deliver.
D10	Socio-political context	24	Socio-political Context	The government provides sufficient reimbursement to the standardised care packages.
		25	Socio-political Context	Primary Health Care is sufficiently oriented towards the delivery of standardised care packages.
D12	Patient	26	Patient characteristics	Patients are positive about the standardised care packages.
D13	Innovation strategy	27	Innovation strategies	There have been provided sufficient materials about the standardised care packages.
D17	Behavioural regulation	28	Action planning	I have a clear plan for when I will deliver the standardised care packages.
D18	Nature of the behaviours	29	Automaticity	Delivering the standardised care packages is something I do automatically.
		30	Memory	Delivering standardised care packages is something I seldom forget.

Distribution of responses to individual items of the modified DIBQ

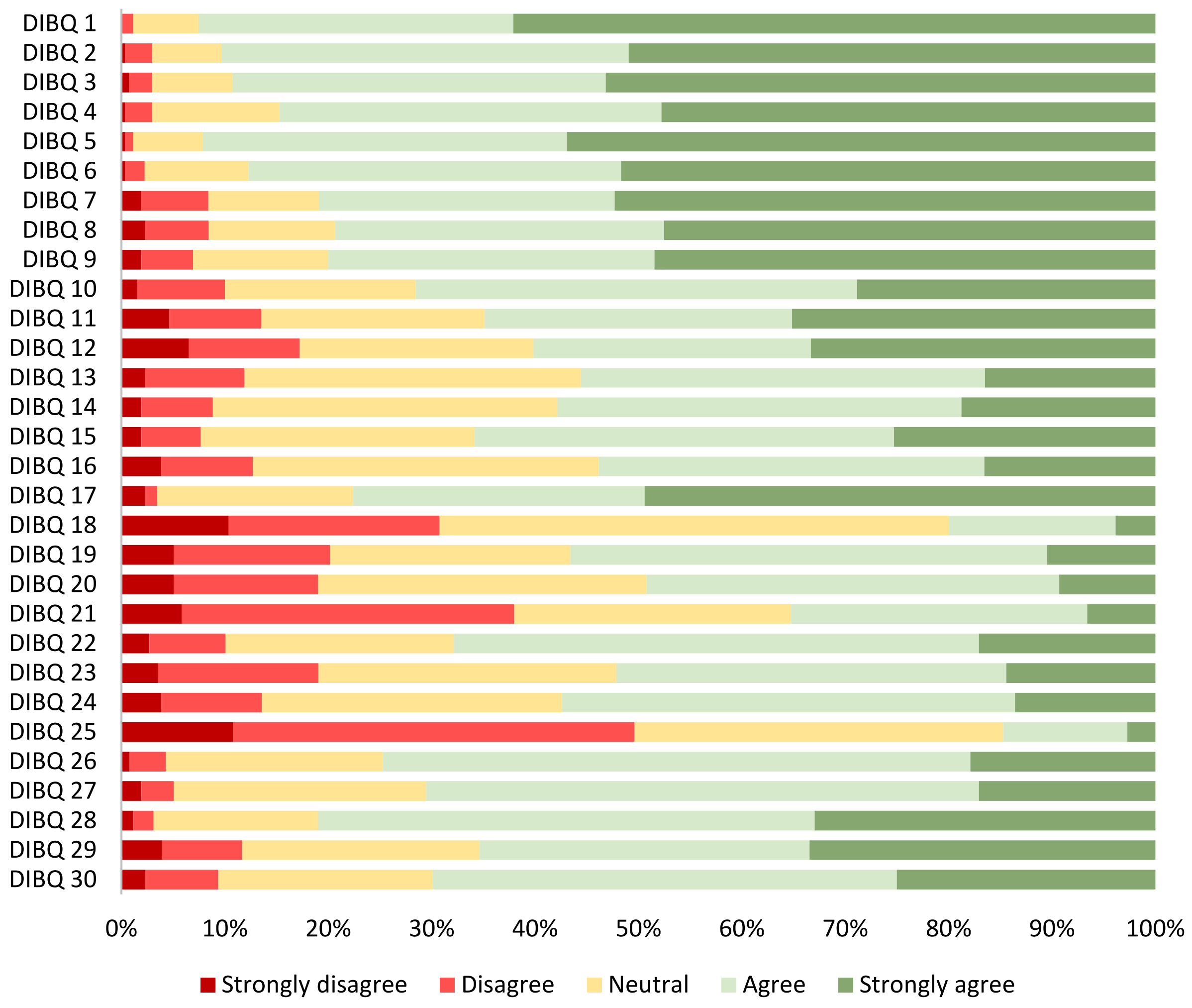

Supplement: Supplementary file 2 — Additional file 2: Items of DIBQ questionnaire, related domains, and distribution of responses to individual items. [file 12998_2022_423_MOESM2_ESM.pdf]
